# The emerging role of sacubitril/valsartan in pulmonary hypertension with heart failure

**DOI:** 10.3389/fcvm.2023.1125014

**Published:** 2023-05-18

**Authors:** Yu Xu, Bowen Yang, Jingjiao Hui, Cai Zhang, Xiaoyun Bian, Min Tao, Yipeng Lu, Wei Wang, Hui Qian, Zhenglu Shang

**Affiliations:** Department of Cardiology, Wuxi Huishan District People's Hospital, Wuxi, China

**Keywords:** sacubitril/valsartan, pulmonary hypertension, pharmacological mechanism, clinical benefits, left heart failure, right ventricular dysfunction

## Abstract

Pulmonary hypertension due to left heart disease (PH-LHD) represents approximately 65%–80% of all patients with PH. The progression, prognosis, and mortality of individuals with left heart failure (LHF) are significantly influenced by PH and right ventricular (RV) dysfunction. Consequently, cardiologists should devote ample attention to the interplay between HF and PH. Patients with PH and HF may not receive optimal benefits from the therapeutic effects of prostaglandins, endothelin receptor antagonists, or phosphodiesterase inhibitors, which are specific drugs for pulmonary arterial hypertension (PAH). Sacubitril/valsartan, the angiotensin receptor II blocker-neprilysin inhibitor (ARNI), was recommended as the first-line therapy for patients with heart failure with reduced ejection fraction (HFrEF) by the 2021 European Society of Cardiology Guidelines. Although ARNI is effective in treating left ventricular (LV) enlargement and lower ejection fraction, its efficacy in treating individuals with PH and HF remains underexplored. Considering its vasodilatory effect at the pre-capillary level and a natriuretic drainage role at the post-capillary level, ARNI is believed to have a broad range of potential applications in treating PH-LHD. This review discusses the fundamental pathophysiological connections between PH and HF, emphasizing the latest research and potential benefits of ARNI in PH with various types of LHF and RV dysfunction.

## 1. Introduction

Exercise intolerance and a loss of compensatory mechanisms are prevalent in advanced pulmonary hypertension (PH). In patients with advanced left heart failure (LHF), PH may persist for an extended period and serve as a marker of poor prognosis. Small pulmonary artery remodeling results in increased pulmonary vascular resistance (PVR) and pulmonary artery pressure (PAP). The right ventricle is particularly susceptible to pressure overload, and it can no longer maintain cardiac output through hypertrophy and increased contractility (1, 2). Over the past decade, PH and right ventricular (RV) dysfunction have attracted significant interest in LHF (3, 4). However, the effective treatment for these complex diseases remains elusive, necessitating further research into prospective medications.

Initially, activation of the renin-angiotensin-aldosterone system (RAAS) contributes to increased blood pressure and cardiac contractility, but it later exacerbates HF due to fluid retention. Overactivation of RAAS also promotes pulmonary vascular and RV remodeling by stimulating cell proliferation, hypertrophy, and vasoconstriction (5). Pulmonary vascular remodeling is mitigated by treatment with angiotensin receptor antagonists (ARBs) (6). Angiotensin-converting enzyme inhibitor (ACEI) or ARB treatment demonstrated favorable safety and tolerability profiles in patients with PAH, and these individuals also experienced lower rates of HF re-hospitalization (7). To counteract the negative consequences of aberrant RAAS activation, the body releases a group of vasoactive peptides known as natriuretic peptides (NP) (8). Atrial NP (ANP), B-type NP (BNP), and C-type NP (CNP) have been found to protect against PH progression. ANP and BNP infusion improved human lung hemodynamics, while CNP improved PH in experimental rats (9, 10). ANP and BNP promote vasodilation and prevent vascular remodeling, counteracting the deleterious effects of RASS on the heart. They also exert anti-proliferative effects on pulmonary vascular smooth muscle cells (11, 12). Despite these advancements in mitigating PH progression with HF, single drug preparations have not yielded more promising therapeutic effects on this condition.

Sacubitril/valsartan, known as ARNI, rectifies the imbalance between the RAAS and NP systems, exhibiting significant efficacy in LHF (13). Through simultaneous inhibition of neprilysin and the angiotensin AT1 receptor, ARNI suppresses pro-fibrotic/pro-hypertrophic mechanisms while promoting anti-fibrotic/anti-hypertrophic mechanisms (14). Prostanoids, endothelin receptor antagonists, or Ca^2+^ channel blockers have been used to treat PH, but the 5-year survival rate remains below 60% (15). Echocardiographic parameters, including LVEF, systolic PAP, and cardiac valvular insufficiency, consistently improved after ARNI therapy (16). ARNI exerts anti-toxicity and vasodilation effects by enhancing the cGMP signaling pathway and inhibiting NP degradation. Furthermore, ARNI remains effective when other vasodilators fail to reverse PH. By increasing the pulmonary artery pulsatility index and decreasing PVR, ARNI enhanced RV-PA coupling, cardiac index, and left ventricular (LV) function (17). This article focuses on the latest developments of ARNI in the treatment of PH, particularly when associated with LHF and RV dysfunction, as this is a novel and underexplored area.

## 2. Pharmacological mechanism of sacubitril/valsartan

Coronary artery disease and hypertension contribute to LV systolic dysfunction, instigating sustained pathological activation of the RAAS and SNS (8). NPs are released to counterbalance atrial and ventricular dilatation in response to RAAS and SNS functional impairment. ANP, BNP, and CNP possess natriuretic, diuretic, vasodilatory, antifibrotic, and antihypertrophic properties (18). However, their role in HF is overshadowed by the vasoconstriction and sodium-retaining capacity of RAAS.

NP cleavage is primarily catalyzed by the neutral endopeptidase neprilysin (NEP). NEP inhibition elevates bradykinin, NP, and adrenomedullin levels, mitigating the neurohormonal activation that leads to sodium retention, vasoconstriction, and cardiac remodeling (19). NEP is not solely involved in NP catabolism but also participates in the degradation of other bioactive peptides, such as adrenomedullin, endothelin, substance P, and angiotensin II (Ang II). Although NEP inhibition alone increases NP levels, this effect might be counteracted by a concomitant rise in Ang II and other peptides (20). Although the diuretic and natriuretic effects of NEP inhibitors are not linked to harmful RAAS activation, oral administration of the precursor drug candoxatril does not result in a sustained antihypertensive effect. The absence of a blood-pressure-lowering effect from NEP inhibitors is secondary to NEP catabolism inhibition, which raises Ang II and endothelin 1 (ET-1) levels, neutralizing the enhanced vasodilatory effect of NEP inhibition (21, 22).

Sacubitril/valsartan is a first-in-class ARNI composed of valsartan's molecular portion and the NEP inhibitor prodrug, sacubitril. In the Prospective Comparison of ARNI with ACEI to Determine Impact on Global Mortality and Morbidity in Heart Failure (PARADIGM-HF), it was observed that ARNI increased BNP and cGMP levels through NEP inhibition (23). Blockade of the type 1 Ang II receptor (AT1R) inactivated multiple tyrosine-phosphorylated proteins responsible for cell proliferation, hypertrophy, and fibrosis, including the JAK kinase family (JAK2 and Tyk2) and phosphorylated kinase-C (PKC). Elevated NP levels also produced favorable biological effects via the soluble guanosine cyclase (sGC)/cGMP pathway (24, 25). The enzyme PKG, which mediates titin phosphorylation, experienced further enhancement by cGMP. Patients with heart failure with preserved ejection fraction [HFpEF, defined as left ventricular ejection fraction (LVEF) ≥ 50%] exhibited a high ratio of stiff (N2B) isoforms to compliant (N2BA) isoforms. Phosphorylation of N2B isoforms by PKG reduced resting stiffness of cardiomyocytes (14). Furthermore, during a follow-up period lasting 12 weeks after discharge, the levels of NT-proBNP and the risk of endpoint events such as cardiovascular death and rehospitalization for HF were reduced by an average of 30% in patients with HF who continued taking ARNI compared to those who switched to enalapril (26). Matrix metalloproteinase (MMP)-9 levels, along with its specific inhibitor, tissue inhibitor of metalloproteinase levels (TIMP)-1, and the levels of procollagen amino-terminal prepropeptide type I (PINP) and type III (PIIINP), were reduced following ARNI treatment, indicating a decrease in collagen fibers (27).

MicroRNAs (miRs) play a role in regulating cardiac apoptosis, angiogenesis, fibrosis, and myocardial hypertrophy, leading to molecular and structural adaptive changes that could impact HF pathology (28). In a rodent model of chronic myocardial infarction, ARNI treatment led to the downregulation of miR-181a expression, which in turn attenuated myocardial fibrosis and pathological hypertrophy (29). After one year of follow-up in cardiac resynchronization therapy with a defibrillator (CRTd) non-responders, patients treated with ARNI exhibited elevated levels of miR-18 and miR-145, and decreased levels of miR-181. Indirect evidence of the advantageous epigenetic effects of ARNI in high-risk failing patients is demonstrated by the direct correlation between plasma miR-18 and miR-145 fold increases with EF improvements, and the inverse correlation with NT-proBNP (30).

## 3. The RAAS and NPs in PH

When the circulating blood volume (due to blood loss or dehydration) or cardiac output decreases, juxtaglomerular cells situated on the lateral endothelium of the afferent arterioles release renin into circulation. The primary function of renin is to hydrolyze angiotensinogen, secreted from the liver, to produce angiotensin I (Ang I). In pulmonary artery endothelial cells, ACE cleaves Ang I to Ang II by removing two C-terminal residues. Ang II upregulates vasopressin released from the central nervous system and induces contraction of vascular smooth muscle cells in the pulmonary circulation, as well as in systemic arterial and venous circuits (31).

Indeed, the RAAS is more complex than the classical pathway. Ang II may be produced by chymotrypsin found in mast cells and skeletal muscle or by cathepsin G present in inflammatory cells (32). The binding of Ang II to AT1 receptors causes vasoconstriction by upregulating ET-1 or decreasing NO bioavailability. Stimulation of AT1 receptors leads to the migration and proliferation of vascular smooth muscle cells, as well as cardiomyocyte hypertrophy (33). ACE2, a homolog of ACE, competes with ACE1 to convert Ang II to Ang 1–7 and Ang 1–9. The ACE2/Ang1–7/Mas receptor (MasR) axis is known to provide cardioprotective effects. In type-2 diabetic (T2DM) patients with poor glycemic control, myocardial levels of both ACE2 and glycosylated ACE2 were elevated, while the expression of Ang 1–9, Ang 1–7, and MasR was reduced, indicating impaired ACE2 activity and the anti-remodeling effects of renin-angiotensin system (RAS) suppression. High levels of myocardial fibrosis were subsequently observed in these patients and in T2DM explanted hearts (34, 35). Ang (1–7) can be produced not only by cleaving a carboxylate-terminal residue from Ang II via the carboxypeptidase ACE2, but also directly from Ang I by NP and prolyl-carboxypeptidase. Ang (1–7) binding to the G protein-coupled MasR counteracts Ang II to produce vasodilatory effects without stimulating aldosterone secretion (36, 37). Plasma renin activity, Ang I, and Ang II levels were significantly elevated in patients with PAH, which were positively related to disease deterioration and markedly increased the risk of death or lung transplantation. The arterial hypertension group of rats receiving Ang II infusion exhibited atrial and perivascular fibrosis in the aorta and pulmonary arteries, with increased AT1 receptor binding in the great vessels but unchanged atria. This suggests that these responses were not related to ventricular wall stress but to RAAS hormonal effects (38). In line with this, RAAS promoted the proliferation of pulmonary artery smooth muscle cells through increased AT1 receptor binding in patients with idiopathic PAH (iPAH) (39). In a piglet overflow model, losartan resulted in a 51% and 35% reduction in shunt-induced PVR and medial thickness, respectively. Decreased PVR was accompanied by a sustained increase in ET-1, ETB receptor, and Ang1 expression, suggesting that Ang II antagonists and ET receptor blockers could be combined in early PAH (40). Ang II predominantly binds to angiotensin type 1 receptor (AGTR1) to promote vascular smooth muscle contraction. Chung WK et al. discovered a correlation between AGTR1 and age at iPAH diagnosis, while no such correlation was found with AGT, ACE, CMA1, or CYP11B2. This finding suggests that losartan may play a role in delaying PAH disease progression (41).

**Figure 1:**
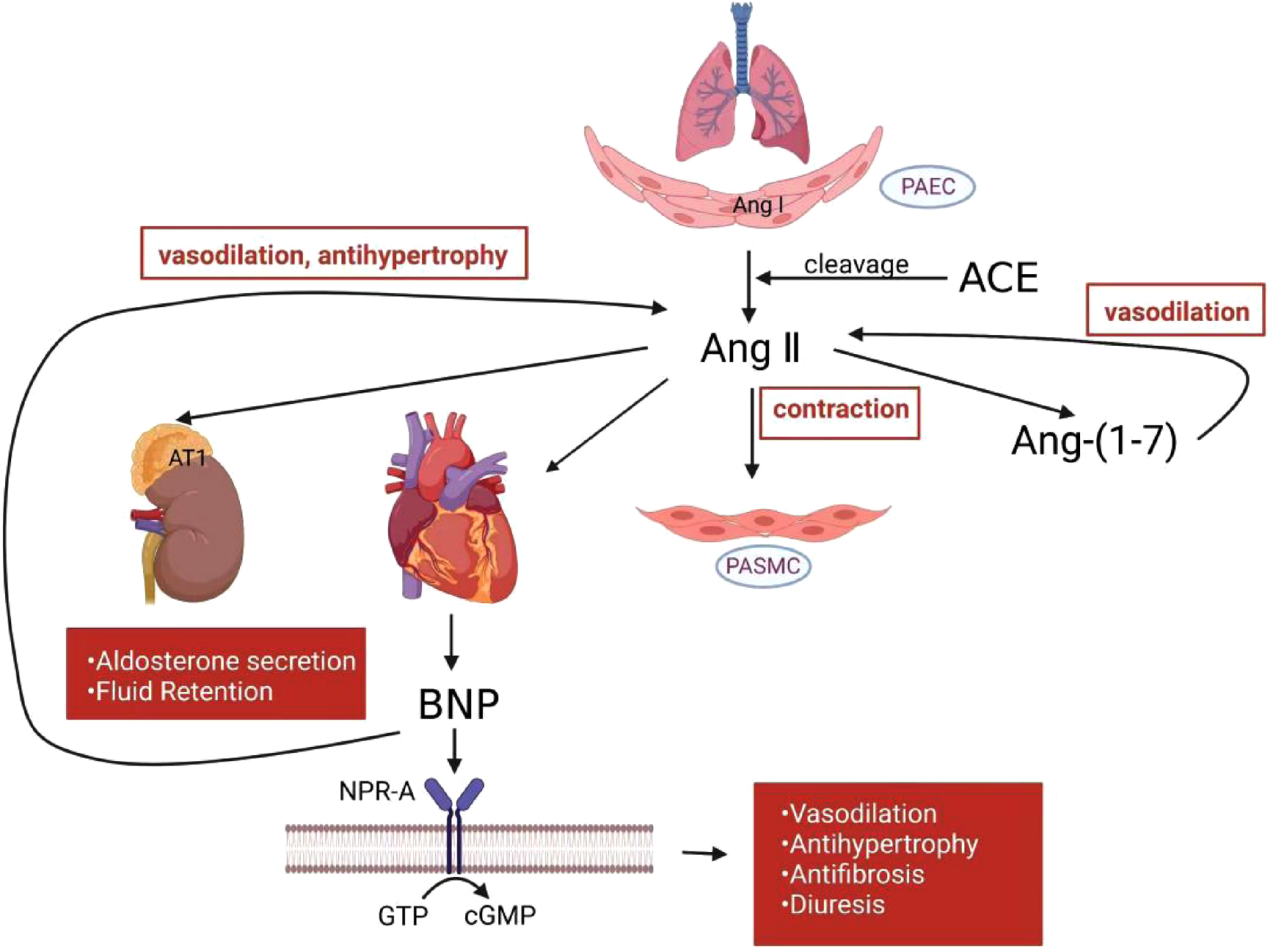
RAAS and NP mechanism in PH. In the pulmonary circulation, activated RAAS induce smooth muscle cell contraction. Angiotensin-(1–7) counteracts angiotensin II to produce vasodilatory effects. BNP released from heart ventricle counteracts RAAS to attenuate vasoconstriction, hypertrophy, fibrosis and other deleterious effects. RAAS, renin-angiotensin-aldosterone system; NP, natriuretic peptide; PH, pulmonary hypertension; PAEC, pulmonary artery endothelial cells; PASMC, pulmonary artery smooth muscle cell; Ang I, angiotensin I; Ang II, angiotensin II; Ang-(1–7), angiotensin-(1–7); ACE, angiotensin-converting enzyme; BNP, B-type natriuretic peptide; NPR-A, natriuretic peptide receptor-A; GTP, guanosine triphosphate; cGMP, cyclic guanosine monophosphate.

Pulmonary vascular remodeling is characterized by hyperplasia of the media and neo-muscularization of the subendothelial layer, leading to vasodilatory dysfunction, arterial lumen narrowing, and elevated PAP. ANP and BNP have been implicated in the pathogenesis of myocardial hypertrophy and fibrosis (42). BNP, though primarily triggered by cardiomyocyte stretch, is upregulated in PH (43). By activating the particulate guanylyl cyclase-linked receptor and natriuretic peptide receptor-A (NPR-A), BNP traditionally mediates vasodilatory effects to increase intracellular cGMP levels (44). (Figure 1) Wijeyaratne CN et al. found that BNP also inhibits vascular smooth muscle proliferation and counteracts the RAAS, thereby attenuating pulmonary vascular remodeling and inhibiting the synthesis of growth factors such as endothelin (45). Infusion of the human BNP nesiritide decreased right atrial pressure, mean PAP, and post-pulmonary capillary wedge pressure (PCWP) in patients with HF and PH, thus increasing cardiac output (46). The vasodilator nitric oxide (NOx) levels were reduced in patients with PH, but the expression of NOx receptors and ET-1 was increased. Nesiritide significantly increased NOx and cGMP levels to promote vasodilation in these patients (47). Nesiritide may have therapeutic potential to slow the progression of RV dysfunction. Nesiritide rapidly reduced PCWP, and increased stroke volume and cardiac output after administration for 3 h, with these effects persisting for at least 24 h (48). Although BNP alone had no significant effect on pulmonary hemodynamics, it enhanced the diastolic effect of the phosphodiesterase-5 inhibitor sildenafil on the pulmonary vasculature (44).

## 4. Sacubitril/valsartan in PH with different types of LHF

The 5th World Symposium on PH proposed classifying PH into five categories: (1) PH due to pulmonary vascular disease; (2) PH due to LHD; (3) PH due to lung disease or hypoxia; (4) PH due to chronic thromboembolic disease; (5) a miscellaneous collection of PH syndromes caused by a variety of disorders, including hemolytic anemias and sarcoidosis (49).

### 4.1. Characterization of PH in HFrEF and HFpEF

LHD is one of the most common causes of PH and is typically classified as post-capillary or group 2 PH. Hemodynamic parameters that predict survival and prognosis in patients with HF include pulmonary artery wedge pressure (PAWP), mean PAP and PVR, and PA compliance/capacitance (50, 51). In post-capillary PH, an elevation of PAWP leads to a corresponding increase in mean PAP to maintain an average transpulmonary pressure gradient (TPG = mPAP—PAWP) of less than 12 mm Hg (52). However, TPG is influenced by volume loading and cardiac function, which does not predict prognosis in PH-LHD. The diastolic pressure gradient (DPG), determined by the difference between diastolic PAP and PAWP, is thought to be less dependent on stroke volume and loading conditions. Intimal hypertrophy, intima and outer membrane fibrosis, and vascular occlusion were observed in patients with pre- and post-capillary (Cpc) -PH and with DPG ≥ 7 mmHg, suggesting that decreased pulmonary vascular compliance may also cause small vessel proliferative vasculopathy (53, 54). However, patients with PH-LDH and with DPG ≥ 7 mm Hg had a lower survival rate than those with DPG < 7 mmHg, and DPG was only minimally predictive of idiopathic cardiomyopathy (55). By applying the support vector machine–recursive feature elimination algorithm, our group found that EPB42 and IFIT2 were highly expressed in PAH patients, while FOSB and SNF1LK showed opposite trends. These four potential genes may distinguish PH patients from healthy individuals and can be used for early diagnosis of PH (56).

Up to 60% of patients with severe LV systolic insufficiency and 70% of patients with pure LV diastolic insufficiency may develop PH (57, 58). There were also differences between PH-HFrEF [heart failure with reduced ejection fraction, defined as left ventricular ejection fraction (LVEF) < 40%] and PH-HFpEF. In a report combining retrospective and prospective data, using DPG > 7 mmHg as a diagnostic criterion, the Cpc-PH values for HFpEF and HFrEF were 22.6% and 18.8%, respectively (59). For similar DPG cutoff values, the Cpc-PH rate observed in PH-HFpEF was more than two times higher than that of PH-HFrEF (60). However, patients with stage D HFrEF may also have a Cpc-PH-LHD phenotype, with specific manifestations of mean PAP > 25 mm Hg, PCWP > 15 mm Hg, DPG > 7 mm Hg, and PVR > 3 WU (61). In HFrEF and HFpEF, the initiating factor for PH is impaired LV diastolic and filling function, which allows elevated left atrial pressure to reach the right heart eventually. The type of LV cardiomyocyte hypertrophy and the amount of reactive and alternative fibrosis well distinguished between HFpEF and HFrEF (62). HFrEF is more prevalent in conditions such as ischemic cardiomyopathy, dilated cardiomyopathy, and secondary mitral valve insufficiency. The primary mechanisms of HFrEF formation are cardiomyocyte elongation and loss of LV compliance. Impaired left atrial kinetics form the basis of PH elevation. HFrEF predominantly presents with increased left atrial and eccentric remodeling as a result of severe mitral regurgitation. Notably, in patients with HFrEF and LV dilatation, functional mitral regurgitation is common and may be a potentially significant cause of PH. In these patients, mitral valve repair treatment has been shown to significantly improve pulmonary hemodynamics, including reductions in mean PAP and PAWP (63, 64). Patients with hypertension, obesity, and diabetes mellitus are susceptible to HFpEF, which leads to LV centripetal hypertrophy and increased diastolic stiffness. Even during the early stages of HFpEF, unfavorable diastolic ventricular interactions have been observed during exercise, serving as the primary mechanism of increased PAWP in the obese phenotype (65).

### 4.2. Clinical application of sacubitril/valsartan in PH with HFrEF and HFpEF

A retrospective cohort study discovered that patients with HFrEF exhibited a significant decrease in pulmonary artery systolic pressure (PASP) after six months of early initiation of treatment with ARNI, which, in conjunction with LV reverse remodeling, demonstrated a better prognosis. This effect of ARNI does not appear to be dependent on other medications (66). Real-world studies observed that ARNI reduced LV end-systolic volume and systolic PAP in patients with HFrEF over six months, even with a reduced furosemide dosage (67). Gender differences were also observed in the effect of initial ARNI treatment on PAP. At up to 12 months, LVEF, relative wall thickness, and E/A did not show greater improvement in women than in men, but left atrial diameter and PAP demonstrated superiority in women (68). As a new first-line agent in HF, patients with HFrEF taking ACEI or ARB experienced a rapid decrease in PAP after switching to ARNI, which was equally effective in patients with low TPG, relatively normal PVR, and elevated TPG and (or) PVR (69).

Pulmonary artery stiffness (PAS) is a crucial determinant of pulmonary sclerosis, characterized by increased vascular stiffness, pulmonary artery endothelial dysfunction, and inflammation (70). New non-invasive tools such as echocardiography and cardiac magnetic resonance imaging (MRI) can quantify PAP and vascular resistance (71). PAS was significantly elevated in patients with HFrEF and was independently associated with the severity of the New York Heart Association (NYHA) functional class. A recent study demonstrated that after six months of ARNI administration for patients with HFrEF, there was a significant decrease in PAS calculated from the maximal frequency shift and acceleration time of the pulmonary artery flow trace. The study also showed a significant improvement in LV remodeling, RV function, and exercise tolerance (72).

Prospective Comparison of ARNI With ARB Global Outcomes in HF With Preserved Ejection Fraction (PARAGON-HF) is a multicenter, international, randomized, double-blind, event-driven trial designed to compare the long-term efficacy and safety of ARNI vs. valsartan alone in patients with chronic HF with LVEF >45%. The prevalence of PH in patients with HFpEF was close to one-third, and elevated PASP might serve as an independent risk factor for predicting mortality in patients with HFpEF in the trial (73). In HF hospitalized patients with significantly elevated PASP, HFpEF-PH had a higher 5-year mortality rate, possibly because several therapeutic regimens have been used to reduce HFrEF mortality. A subset of patients with HFpEF appeared to develop intrinsic pulmonary vascular disease, evidenced by elevated mPAP and an increase in PVR and TPG (74). Recent studies have shown that ARNI was equally effective in patients with HFpEF-PH, and even the lowest dose of ARNI significantly reduced PAP and mean PCWP in patients with HFpEF. Not only were the hemodynamic parameters improved, but the NYHA functional class was also enhanced by at least one level (75) (Table 1).

**Table 1:** Effects of sacubitril/valsartan on PH in patients with HFrEF and HFpEF.

Type of experiment	Type of HF	Results	Reference
Retrospective cohort study	HFrEF	Improvement of LV reverse remodeling Reduction of PASP	M.G. Moon, et al. (2021)
	HFrEF	Improvement of PAP and LA diameter (women > men)	M. Landolfo, F, et al. (2020)
Retrospective case-series	HFrEF	A rapid decrease in PAP after switching to sacubitril/valsartan	J.S. Tran, et al. (2021)
	HFpEF	Reduction of PAP and mean PCWP Improvement of NYHA function	Burgdorf C, et al. (2021)
Prospective observational study	HFrEF	Reduction of LV end-systolic volume and systolic PAP	M.V. Polito, et al. (2020)
Cross-sectional,retrospective, and single center study	HFrEF	Reduction of PAS Improvement of LV remodeling, RV function, and exercise tolerance	Yenerçağ M, et al. (2021)
Case report	HFrEF	Improvement of LV systolic function Reduction of PAH	Gulin D, et al. (2019)

HFrEF, heart failure with reduced ejection fraction; HFpEF, heart failure with preserved ejection fraction; LV, left ventricular; RV, right ventricular; PASP, pulmonary artery systolic pressure; LA, left atrial; PAP, pulmonary arterial pressure; PCWP, pulmonary capillary wedge pressure; PAS, pulmonary artery stiffness; NYHA, New York heart association; PH, pulmonary hypertension; PAH, pulmonary arterial hypertension.

## 5. Implications of sacubitril/valsartan for PH with right ventricular dysfunction

Elevated mean PAP is not sufficient to define pulmonary vascular disease, as the causes for PH can vary, including increased cardiac output, elevated pulmonary wedge pressure, and hyperviscosity. At the 6th World Symposium on PH, it was also recommended that DPG should be excluded from the definition of Cpc-PH as it is not necessarily a poor prognostic factor (76). Thus, it remains controversial whether DPG is important to LHD-PH prognosis because the underlying diseases causing LV dysfunction might be different. Impairment of RV contractile function and increased afterload due to PAH may both contribute to RV dysfunction (77).

Chronic right heart failure (RHF) results from a long-term increase in RV afterload that eventually overwhelms the compensatory mechanisms of the RV. Epinephrine stimulates the compensatory drive mechanisms that maintain systolic cardiac function when RV afterload is increased but ultimately leads to myocardial dysfunction if sustained over time (33). Furthermore, the prolonged increase in adrenergic tone results in downregulation of RV myocardial beta receptors and depletion of norepinephrine reserves (78). A recent nuclear imaging study of a small group of patients with PAH suggested these patients might have significant sympathetic dysfunction. Compared with the control group, the Cardiac (79) Iodine-metaiodobenzylguanidine uptake showed reduced LVEF in the PH group. Heart-to-mediastinum ratios and washout rate are associated with PVR, right atrial pressure, tricuspid plane systolic excursion, NT-proBNP, and peak VO_2_ (80). In a rabbit model of systemic RV afterload induced by pulmonary artery band, ventricular-ventricular interactions via TGF-b1, CTGF, and ET-1 signaling pathways led not only to RV hypertrophy but also to secondary LV fibrosis and RV apoptosis. However, Ang-II receptor blockade with losartan ameliorated this interventricular crosstalk. Notably, LV CTGF mRNA expression increased after pulmonary artery band treatment, and losartan reduced pulmonary artery band-induced CTGF mRNA expression (81). Since pulmonary RAAS activity correlates with prognosis in patients with iPAH, it can be hypothesized from this experiment that losartan may reverse RV hypertrophy and reduce LV load by blocking pro-fibrotic signals. Telmisartan has also been found to improve RV remodeling, possibly through inhibition of MMP-2 and MMP-9 activity (82). However, Borgdorff MA et al. discovered that losartan combined with eplerenone did not improve RV systolic and diastolic function, nor did it prevent myocardial fibrosis and RV hypertrophy (83). The reason for this contrast may be the physiological difference between LV and RV, or inhibition of RAAS secondary to the blockade of AT1R on the pulmonary vasculature, rather than the direct myocardial effect on RV. Doppler cardiac ultrasound and cardiopulmonary exercise testing revealed that losartan reduced PAP and right atrial diameter, and improved patients’ exercise tolerance (84). Differences in the physiology and complex pathologic structure of human and rodent hearts may account for the issue. More robust evidence from invasive tests (placement of cardiac catheters) is required to measure accurate pressure on the right side of the heart.

RV dysfunction signifies HF progression and may even become a worse prognostic factor (85). In a comparison with the independent RV failure model induced by pulmonary trunk banding, ARNI significantly reduced RV systolic pressure (RVSP), RV hypertrophy, RV end-diastolic, and end-systolic volumes in the group with PH. This suggests that ARNI may not directly affect RV remodeling but produces right heart benefits by improving pulmonary vascular function (86). RV hypertrophy transmural reorientation of collagen and myofibers was also weakened, indicating that the effect of ARNI on RV remodeling was manifested not only in hemodynamics but also in the biomechanical properties of the RV at the tissue level (87). ARNI alone may decrease PAP and RV remodeling through an increase in endogenous NP. Clements RT et al. treated the PH rat model induced by SU5146 and hypoxia with ARNI for six weeks and found a decrease in RV pressure and fibrosis, accompanied by an increase in pulmonary ANP, BNP, and cGMP levels (88). Bosentan, a specific and competitive dual endothelin receptor blocker with low molecular weight, is the first novel oral drug approved for treating PAH. However, Bosentan acts primarily by dilating the pulmonary vasculature without acting directly to prevent RHF. In the monocrotaline-induced rat model of severe PH, ARNI enhanced the effect of Bosentan on reducing PVR, RV hypertrophy, and fibrosis. Cultured human pulmonary artery smooth muscle cells derived from iPAH patients simultaneously validated the anti-smooth muscle proliferative effect of ARNI (89). Thus, ARNI may have a synergistic effect on traditional drugs for treating PH. Loss of the NO pathway typically results in endothelial dysfunction in PH patients, followed by compensatory vasodilation mediated by the natriuretic peptide clearance receptor (NPR-C). Activation of the NPR-C signaling pathway may exhibit antiproliferative effects, while hypoxia-induced downregulation of NPR-C expression may lead to pulmonary vascular remodeling and elevated PAP (90).

Inflammatory infiltration represents another mechanism in the pathogenesis of pulmonary vascular disease, with the accumulation of extracellular matrix proteins such as fibronectin (91). In monocrotaline-induced and hypoxia-induced rats, ARNI not only increased the levels of ANP and CNP in circulating blood and lung tissues but also displayed the same trend in the expression of NPR-A, C, and cGMP. Circulating levels of IL-1β, IL-6, and TNF-α subjected to ARNI intervention were reduced in both animal models, and the anti-inflammatory effect may be related to the ANP/NPR-A/cGMP pathway (92).

## 6. Effects and adverse events of sacubitril/valsartan for PH in advanced HF and chronic kidney disease

Long-term left atrial pressure leads to severe PH in patients with advanced HFrEF, which might disqualify them for heart transplantation. When conventional treatments like diuresis, vasodilation therapy, and mechanical support demonstrated no apparent efficacy, experimental ARNI produced better outcomes than expected. After 24 h of ARNI administration, PASP and PVR were significantly reduced, allowing four patients in real-world cases to regain heart transplant candidacy, without postoperative RHF or hypotension requiring vascular compression support (17).

In addition to HFrEF, adult congenital heart disease patients develop subpulmonary artery ventricular dysfunction and PH. Lluri G et al. reported that four patients with cyanosis, complicated coronary artery disease, and high PAP experienced a significant improvement in symptoms after taking ARNI, and their NYHA class III condition improved to class II (93). A case report observed that one patient developed reduced LVEF and RVSP capacity with PH in the second year after heart transplantation. After practical application of ARNI, this patient exhibited increased activity endurance and improved LV systolic function (LVEF up from 29% to 41%) with a reduction in PAH (RVSP down from 65% to 50%) (94). Even in patients with refractory HFrEF combined with PH, ARNI has shown surprising efficacy. The gradually increasing dose of ARNI reduced filling pressures without impairing normal renal function. With hemodynamic support for a decrease in the right atrial pressure/PCWP ratio, echocardiography displayed a reduction of the E/A ratio and left atrial volumetry (67).

Patients with HF frequently exhibit reduced renal function, while patients with chronic kidney disease (CKD) often face a high risk of cardiovascular events (95). Compared with the RAS inhibitor, ARNI significantly increased estimated glomerular filtration rate (eGFR) and decreased NT-proBNP in patients with both HF and CKD (96). In a real-world study involving patients with all stages of CKD, low baseline GFR <30 ml/min/1.73 m^2^ was identified as an independent predictor for worse clinical outcomes. The data revealed that treatment with ARNI resulted in fewer cardiovascular deaths or hospitalizations for HF than treatment with standard therapy without ARNI in both patients with GFR ≥ 30 ml/min/1.73 m^2^ and with GFR < 30 ml/min/1.73 m^2^ (97). One year of treatment with ARNI significantly improved systolic and diastolic heart function in patients with end-stage kidney disease and HFrEF, but ARNI did not increase hyperkalemia or hypotension risk in these patients (98). Early worsening renal function (WRF), defined as a >20% decrease in eGFR, occurred in patients with ARNI therapy after one month. However, renal function recovered in these patients at three months, with an improvement in eGFR at one year compared with the baseline value. Additionally, early WRF had no impact on clinical outcomes in the following 650 days (99).

By activating both RAAS and NP inhibition, ARNI demonstrates a greater blood pressure reduction than ARB alone (100). In the UK Heart and Renal Protection III (UK HARP III) and PARADIGM-HF trials, hypotension was observed in patients receiving ARNI treatment (101, 102). The occurrence of hypotension is predictable, and only significant symptomatic hypotension leading to pre-syncope, syncope, or other organ damage should justify decreasing the dosing of ARNI (103). Although hyperkalemia was less frequent in the ARNI group than in the enalapril group, it is recommended to check creatinine and serum potassium after ARNI administration (102). The consensus indicates that angioedema is the primary adverse effect leading to the discontinuation of the drug (104), but the incidence of angioedema is rare, and more trials are needed for further investigation. Attention for the risk of Alzheimer's disease (AD) is rising because NEP inhibition may decrease degradation of amyloid-beta (Aβ) protein, which is associated with AD progression (105, 106). NEP1 inhibition made Drosophila Aβ detrimental to both middle-term and long-term memory, while NEP1 overexpression rescued the memory deficits (107). The aforementioned animal models suggest that ARNI may affect cognitive function; however, adverse events related to cognition, memory, and dementia were not elevated in the ARNI group in PARADIGM-HF (108). Further investigation in clinical studies is required to determine whether ARNI will have an impact on cognition in patients with HF and/or CKD.

## 7. Diagnostic tools and monitoring equipment for PH

Transthoracic echocardiography enables the measurement of peak tricuspid regurgitation velocity and the calculation of PASP in assessing RV function (109). Tricuspid annular plane systolic excursion (TAPSE), RV DTI-derived tricuspid lateral annular systolic velocity wave (RV S'), and fractional area change (RV FAC) are recommended echocardiographic parameters for assessing RV systolic function in clinical studies (110, 111). After 12 months of therapy with ARNI in a real-world registry, Correale M et al. reported that improvements in PASP and TAPSE were proportional to baseline levels and independent of LV function (112). Recently, they further demonstrated that the baseline RV S' value is an independent predictor of RV improvement. Peak longitudinal strain of the RV free wall (RV-FW-LS), a more accurate and sensitive tool for evaluating RV function, and RV four-chamber strain (RV-4Ch-LS), a parameter that includes the analysis of the interventricular septum, were also improved in this study (85) (Table 2). Forfia et al. found that TAPSE predicted survival in 47 patients with PAH but not mortality in patients with pre-capillary PH combined with RV dilatation (113). Echocardiography using a multivariable model based on 2D measurements revealed RV dyssynchrony in patients with mean PAP between 20 and 25 mmHg, suggesting that RV deformation can be impaired even in mild/critical PH (114). Cardiac MRI provides access to the 3D structural RV and is recommended as the gold standard for assessing RV end-diastolic and systolic volumes, RV mass, local ventricular wall motion, and pulmonary artery blood flow (115). RV quality has been shown to predict PH prognosis, and both stroke volume index lower than baseline and reduced stroke volume index during treatment were associated with increased mortality (116).

**Table 2:** Effects of sacubitril/valsartan in patients with RV dysfunction.

RV measurements	Type of experiment	Results	Reference
RV end-diastolic and end-systolic volumes	Rat model of RV failure	Reduced	Andersen S, et al. (2019)
RVSP	Rat model of RV failure	Reduced	Andersen S, et al. (2019)
	Rat model of PH	Reduced	Clements RT, et al. (2019)
RVDP	Rat model of PH	Reduced	Clements RT, et al. (2019)
RV maximum pressure	Rat model of PH	Reduced	Sharifi KiaD, et al. (2020)
RVFW thickness	Rat model of PH	Reduced	Sharifi KiaD, et al. (2020)
RV s’	Rat model of PH	Improved	Clements RT, et,al. (2019)
	Real-world study of patients with HFrEF	Improved	Correale M, et al. (2020)
TAPSE	Real-world study of patients with HFrEF	Improved	Correale M, et al. (2020)
	Real-world study of patients with HFrEF	Improved	Correale M, et al. (2021)
RV-FW-LS	Real-world study of patients with HFrEF	Improved	Correale M, et al. (2021)
RV-4Ch-LS	Real-world study of patients with HFrEF	Improved	Correale M, et al. (2021)

RV, right ventricular; RVSP, right ventricular systolic pressure; PH, pulmonary hypertension; RVDP, right ventricular end diastolic pressure; RVWF, right ventricular free wall; RV S’, RV DTI-derived tricuspid lateral annular systolic velocity wave; HFrEF, heart failure with reduced ejection fraction; TAPSE, tricuspid annular plane systolic excursion; RV-FW-LS, peak longitudinal strain of RV free wall; RV-4Ch-LS, RV four-chamber strain.

Patients with end-stage systolic HF are not candidates for heart transplantation due to irreversible PH. However, it can be reversed after 6 months of continuous-flow left ventricular assist device (cfLVAD) implantation (117, 118). CfLVAD reduces PAP and PVR by unloading the left ventricle and lowering LV end-diastolic pressure and volume. Although TPG and PVR return to normal after cfLVAD implantation, histological changes in the pulmonary vascular bed may be irreversible. Differences in DPG gradients between set and maximum velocities >3 mm Hg at baseline cfLVAD levels may indicate persistent capillary PH, which is positively associated with increased HF hospitalization and mortality (119).

CardioMEMS™ is an implantable device positioned in the pulmonary artery to measure cardiac filling pressure in patients with HF, irrespective of LVEF value, which tends to increase more than 2 weeks before symptomatic clinical congestion (120, 121). Pulmonary pressure-guided therapy represents a novel strategy to reduce the risk of recurrence in patients with chronic HF, enabling closer non-invasive in-hospital hemodynamic monitoring using CardioMEMS™. After implanting CardioMEMS™ in a 53-year-old patient with idiopathic dilated cardiomyopathy, the system detected a decrease in PAP, accompanied by an increase in LV ejection fraction and a decrease in NT-pro BNP. This case marks the first reported instance of ARNI improving PAP and cardiac function in a patient with HFrEF, as detected by telemetry data (122, 123). In HFrEF patients with previously implanted CardioMEMS™ sensors, transitioning from ACEI/ARB to ARNI demonstrated a rapid decrease in PAP (69). By monitoring PAP, the CardioMEMS™ system has been shown to balance fluid intake and output, facilitate personalized medication use, and reduce hospitalization rates. After PAP adjustment by CardioMEMS™ to prevent early congestion, ARNI was utilized to decrease the usage of cyclic diuretics, the risk of neurohormonal activation, and electrolyte disturbances (79).

## 8. Conclusions

**Figure 2:**
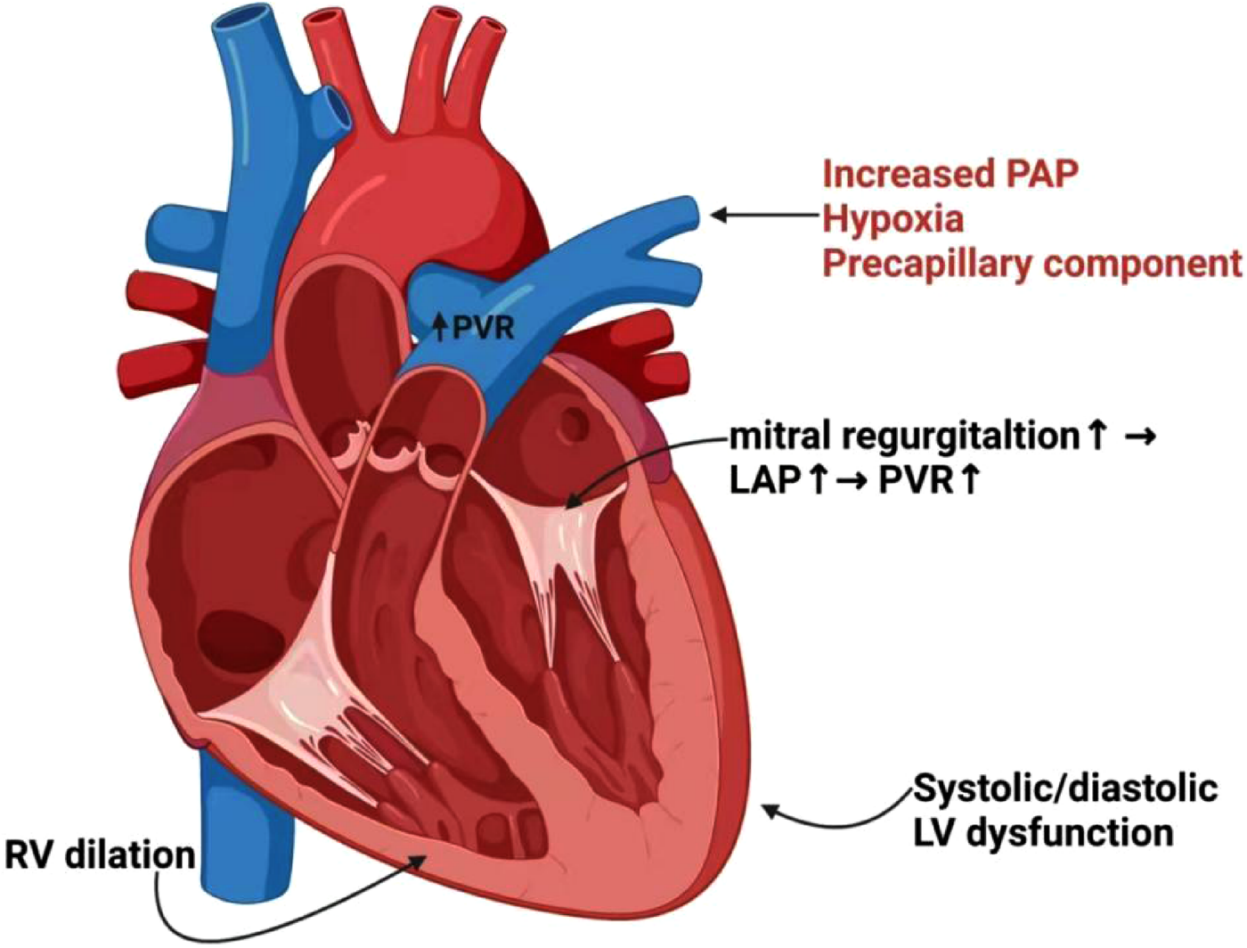
Interaction and pathogenesis of PH in LV/RV heart failure. Hypoxia, precapillary component and increased PAP may trigger pulmonary arterial vasoconstriction, leading to increased PVR and reduced PA compliance. Functional mitral regurgitation will further result in elevations of LAP and PVR. Elevated filling pressures cause PH, which is a consequence of systolic or diastolic LV dysfunction. The persistent elevations of pulmonary pressures and PVR result in dilatation and maladaptive remodeling of right heart chambers, and ultimately RV failure. PH, pulmonary hypertension; PAP, pulmonary artery pressure; PVR, pulmonary vascular resistance; PA, pulmonary artery; LAP, left atrial pressure; LV, left ventricular; RV, right ventricular.

Elevated left heart filling pressures result in impaired pulmonary venous reflux obstruction, which is a primary cause of PH-LHD and ultimately leads to total heart failure (Figure 2). ARNI has become a research focus in the cardiovascular field due to its dual inhibition of the RAAS and NPs systems. In addition to experimental data, evidence from clinical studies suggests that ARNI may be effective in delaying the progression of PH in patients with HFrEF, HFpEF, or RV dysfunction, including those awaiting heart transplantation. Although no serious adverse events have been observed, the future efficacy and safety of ARNI for HF complicated with PH require further large-scale and multicenter clinical studies.
